# *Santalum album* L. alleviates cardiac function injury in heart failure by synergistically inhibiting inflammation, oxidative stress and apoptosis through multiple components

**DOI:** 10.1186/s13020-024-00968-0

**Published:** 2024-07-15

**Authors:** Bojiao Ding, Li Jiang, Na Zhang, Li Zhou, Huiying Luo, Haiqing Wang, Xuetong Chen, Yuxin Gao, Zezhou Zhao, Chao Wang, Zhenzhong Wang, Zihu Guo, Yonghua Wang

**Affiliations:** 1 Key Laboratory of Resource Biology and Modern Biotechnology in Western China, Ministry of Education, Northwest University, No. 229 TaiBai North Road, Xi’an, 710069 Shaanxi China; 2 Jiuwei Institute of Life Sciences, Yangling, 712100 Shaanxi China; 3 Shaanxi Qinling Qiyao Collaborative Innovation Center Co. Ltd., Xianyang, 712100 Shaanxi China; 4 Key Laboratory of Phytomedicinal Resources Utilization, Ministry of Education, Shihezi University, Shihezi, 832000 Xinjiang China; 5 National Key Laboratory On Technologies for Chinese Medicine Pharmaceutical Process Control and Intelligent Manufacture, Jiangsu Kanion Pharmaceutical Co. Ltd., Lianyungang, 222002 Jiangsu China; 6 College of Pharmacy, Heze University, Heze, 274015 Shandong China

**Keywords:** *Santalum album* L., Heart failure, Systems pharmacology, Synergistic effect

## Abstract

**Background:**

Heart failure (HF) is a complex cardiovascular syndrome with high mortality. *Santalum album* L. (SAL) is a traditional Chinese medicine broadly applied for various diseases treatment including HF. However, the potential active compounds and molecular mechanisms of SAL in HF treatment are not well understood.

**Methods:**

The active compounds and possible mechanisms of action of SAL were analyzed and validated by a systems pharmacology framework and an ISO-induced mouse HF model.

**Results:**

We initially confirmed that SAL alleviates heart damage in ISO-induced HF model. A total of 17 potentially active components in SAL were identified, with Luteolin (Lut) and Syringaldehyde (SYD) in SAL been identified as the most effective combination through probabilistic ensemble aggregation (PEA) analysis. These compounds, individually and in their combination (COMB), showed significant therapeutic effects on HF by targeting multiple pathways involved in anti-oxidation, anti-inflammation, and anti-apoptosis. The active ingredients in SAL effectively suppressed inflammatory mediators and pro-apoptotic proteins while enhancing the expression of anti-apoptotic factors and antioxidant markers. Furthermore, the synergistic effects of SAL on YAP and PI3K-AKT signaling pathways were further elucidated.

**Conclusions:**

Mechanistically, the anti-HF effect of SAL is responsible for the synergistic effect of anti-inflammation, antioxidation and anti-apoptosis, delineating a multi-targeted therapeutic strategy for HF.

**Graphical Abstract:**

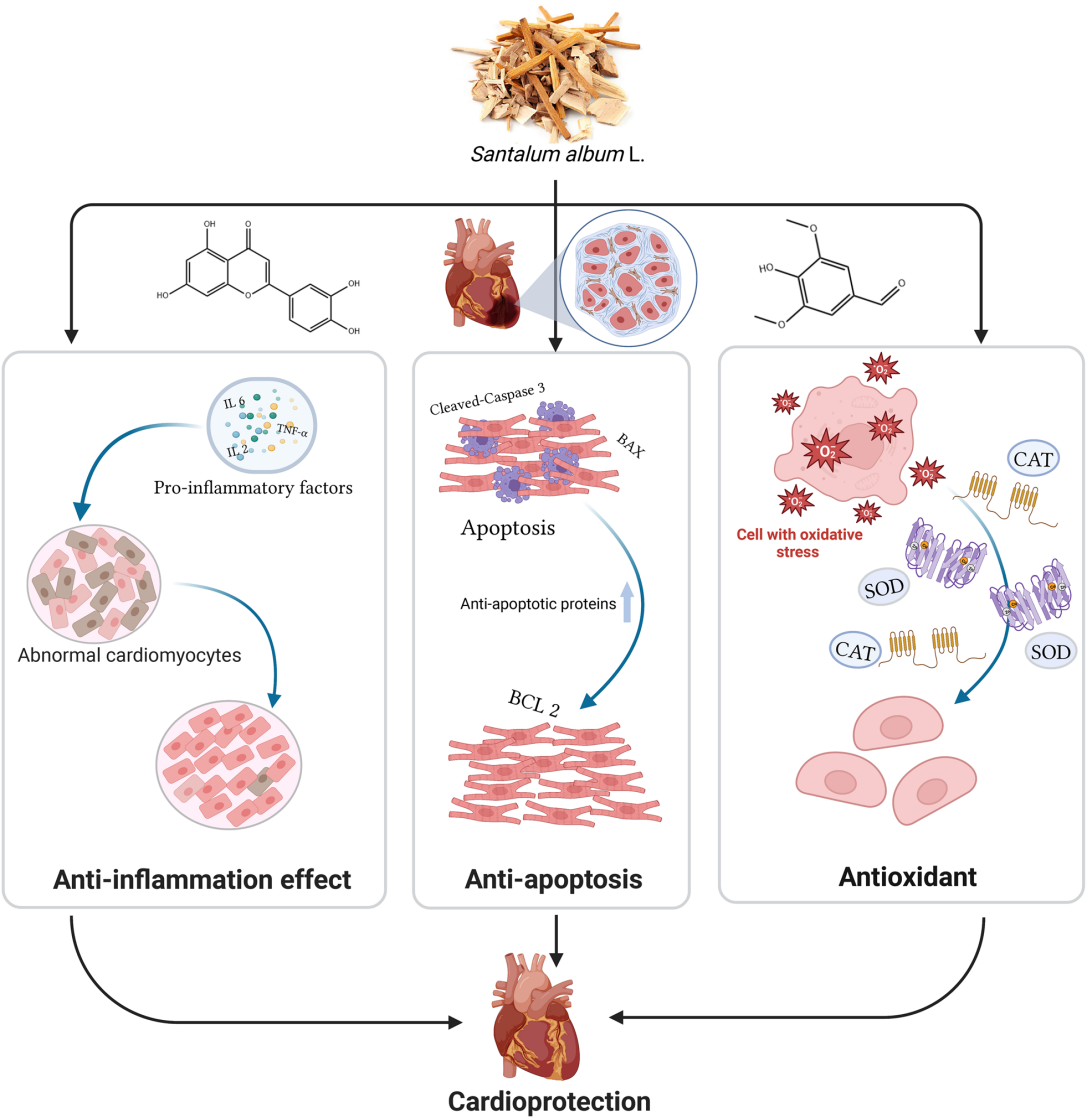

**Supplementary Information:**

The online version contains supplementary material available at 10.1186/s13020-024-00968-0.

## Introduction

Heart failure (HF) is a severe clinical syndrome resulting from various cardiovascular diseases, characterized by changes in the heart muscle such as hypertrophy, inflammation, and fibrosis [1, 2]. Globally, around 38 million individuals suffer from HF, a condition associated with significant morbidity and mortality [3, 4]. Projections indicate a 46% increase in HF incidence by 2030, according to data released by the AHA [5]. Observational data from Europe revealed a 7.2% death rate for chronic stable HF and a 4.1% death rate for acute HF [6]. In China, the prevalence of HF in 2017 reached 1.18% with 248 cases per 100,000 patient-years, alongside a noticeable uptick in outpatient costs in 2019 [7]. Current treatments like hydralazine, nitrate, and aldosterone antagonists have demonstrated improvements in quality of life, symptom reduction, and mortality risk reduction, albeit with potential side effects. Despite advancements in HF survival rates over the past three decades, the 5-year post-diagnosis mortality rate hovers around 50% [8].

Developing new strategies is essential for effective HF management. Traditional Chinese medicine (TCM) has garnered attention in HF treatment, showing fewer side effects compared to Western medications [9]. *Santalum album* L. (SAL), a traditional Tibetan medicine [10], has been utilized in treating various diseases and possesses a variety of medicinal properties [11–14]. A variety of diseases have been clinically treated with SAL's formula for its therapeutic properties and efficacy, including atherosclerosis, viral myocarditis, and acute myocardial infarction [15–17]. Furthermore, SAL exhibits various properties that could help in slowing down the progression of cardiovascular diseases like HF, including antioxidant activity and anti-inflammatory effects [18–21]. Our previous research showed that a saffron-based compound formula improved cardiac function in rats with ISO-induced HF by preventing myocardial hypertrophy and fibrosis [15]. A meta-analysis of clinical trials suggested the potential efficacy of sandalwood in HF treatment [22]. However, due to the complex nature of SAL with multiple components and targets, its molecular mechanism is still not fully understood.

In recent years, the emergence of systems pharmacology has provided a platform to investigate the comprehensive mechanisms of TCM, expediting drug discovery and basic research on herbal medicine. Previous studies have utilized this framework to explore the protective effects of TCM against cardio-cerebrovascular diseases [23, 24]. Consequently, systems pharmacology has also been employed to examine the mechanisms underlying SAL therapy for HF.

**Fig. 1:**
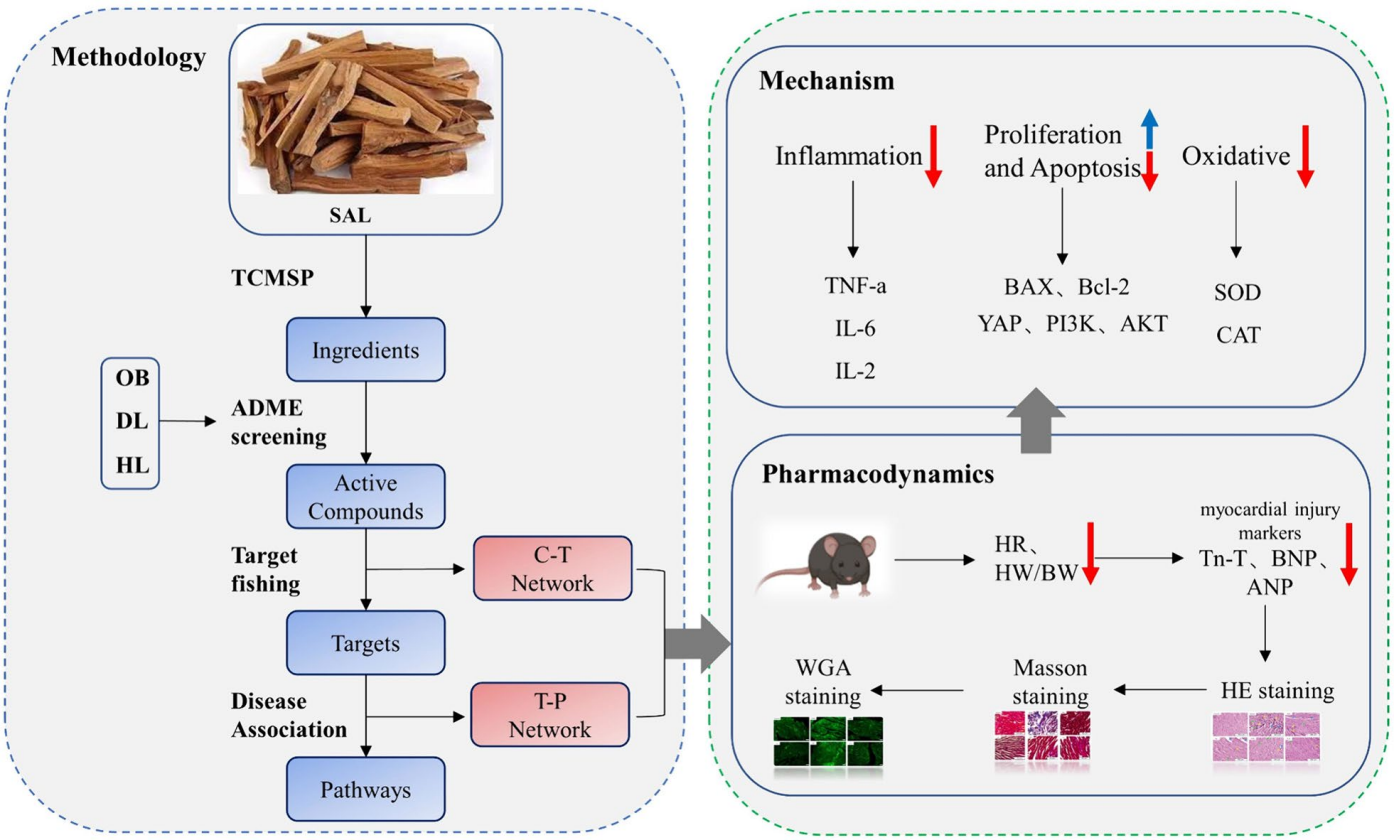
Workflow for SAL alleviates cardiac function injury in heart failure by synergistically inhibiting inflammation, oxidative stress and apoptosis through multiple components

**Fig. 2:**
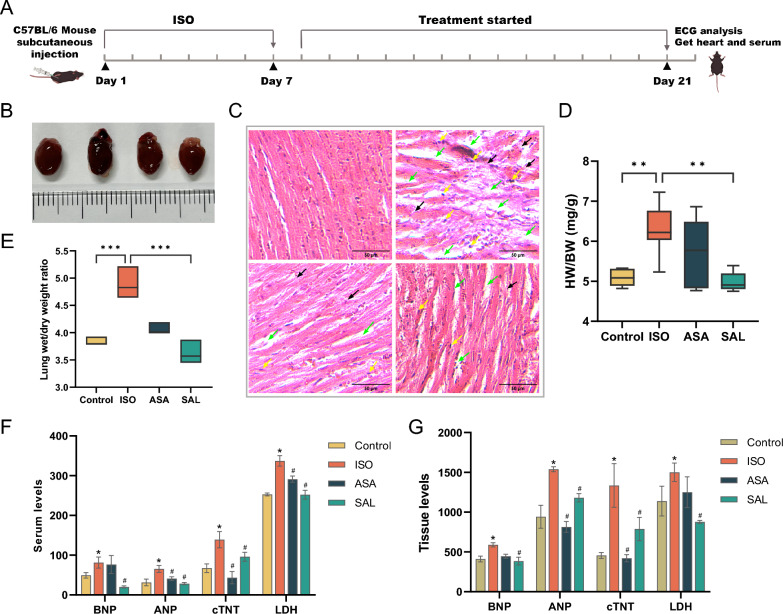
The effect of SAL on myocardial injury in HF (n = 6). **A** The in vivo experimental schema. **B** The appearance of heart. **C** HE staining. Scale bar, 50 μm. Yellow arrow: infiltration of inflammatory cells. Green arrow: rupture and disintegration of the heart muscle fibres. Black arrow: interstitial space of the myocardium. **D** HW/BW (Heart weight/body weight ratio in mice). **E** Lung wet /dry weight. **F** and **G** BNP, ANP, cTNT and LDH levels in serum and heart tissue. BNP, ANP, cTNT, pg/ml. LDH, ng/ml. * a significant difference between ISO group and control group (*P* < 0.05); # displayed significant difference vs ISO group. *ISO* isoproterenol, *SAL Santalum album* L., *ASA* aspirin

To achieve this, we initially evaluated the impact of SAL on heart damage in mice with isoproterenol (ISO)-induced HF. Subsequently, a pharmacokinetic assessment was conducted at the molecular level to identify potential pharmacodynamic compounds and their optimal combinations in SAL. The effectiveness of these compounds was determined through analysis of cardiac function parameters, cardiac injury marker levels, and pathological changes. The therapeutic role of these molecules in treating HF depends on their specific targets. Furthermore, the Weighted Integrated Similarity (WES) and Systematic Drug Targeting Tool (SysDT) methodologies, along with in vivo experiments, were utilized to identify and validate the multiple targets of the bioactive components in SAL. Network analysis at the pathway level was employed to reveal key biological pathways associated with HF and to elucidate the various mechanisms of SAL. Experimental validation was conducted to support the reliability of our approach. Specifically, expression of target proteins in the pathway was evaluated in mice with ISO-induced HF when the active compounds were used alone and in combination (Figs. 1 and 2A). Our findings demonstrate the efficacy of SAL in HF treatment and propose a novel strategy for the discovery of natural drugs.

## Material and method

### Data collection and screening in SAL

All chemical constituents of SAL were retrieved from the TCMSP database (https://old.tcmsp-e.com/tcmsp.php) [25]. Subsequently, three different in silico ADME models incorporating DL (drug-likeness), OB (oral bioavailability) and HL (half-life) respectively were adopted to explore the candidate compounds in SAL (OB ≥ 30%; DL ≥ 0.18 and HL ≥ 4 (long)) [24].

### Prediction of drug combinations and target

The assessment data in this study were analyzed through a probabilistic ensemble aggregation (PEA) method to evaluate and predict the therapeutic and adverse effects of drug combinations in patients. The specific operation mainly involves simulating the combination of drug molecules and pharmacological phenotypes using a Bayesian network. The evaluation was comprehensive, taking into account a variety of determinants including chemical similarity, drug classification, drug side effects, and interactions between target proteins [26–28]. The highest-scoring group was chosen for validation based on the specificity and sensitivity of the predicted results. Additionally, a novel approach (WES and SysDt) was developed to predict the protein targets of SAL for bioactive compounds.

### Biological mechanism of SAL analysis

For exploring the underlying action mechanism of SAL, Gene Ontology (GO) enrichment analysis based on the pharmacological targets was performed. In addition, protein information was mapped to the KEGG database for KEGG pathway analysis.

### Network construction

Two corresponding networks, compound-target (C-T) and target-pathway (T-P) networks were constructed and analyzed in Cytoscape 3.8.0, along with their basic topological properties [29].

### Animals and treatments

Mice (Male, C57BL/6, 18–21 g) were chosen for the in vivo experiments from the Comparative Medicine Centre of Yangzhou University (CMCYU) and Beijing Vital River Laboratory Animal Technology Co., Ltd. in China. The mice were housed in standard conditions with 40–60% relative humidity, 22–25 °C, and 12 h light/dark cycle, with ten mice per cage in our animal room before the commencement of the experiment. The Guide for the Care and Use of Laboratory Animals was followed in all experimental procedures. This study is under the review and approval of the Laboratory Animal Ethics Committee of Jiangsu Kanion Pharmaceutical Co., Ltd. [IACUC No. (2020072003)].

To evaluate the impact of SAL on mice with ISO-induced HF, the mice were randomly divided into four groups: Control group, ISO group, positive group (ASA), and SAL group with 6 mice in each group. ISO (5 mg/kg, CAS# 51-30-9, Meryer, purity ≥ 99%) was administered via subcutaneous injection to the mice in the drug-treated and ISO groups for 7 days. Subsequently, the positive drug group was treated with ASA (aspirin, 15 mg/kg, CAS#50-78-2, Sigma, USA), while the control and ISO groups received an equivalent volume of saline. Aspirin was chosen as the positive control due to its known efficacy in treating ISO-induced HF [30]. The SAL group received SAL extract (48 mg/kg, Cat# 2020100902, Xi'an Mugo Biotechnology Co., Ltd) via for 2 weeks. The dosage of SAL was determined based on previously published studies and our own preliminary experiments [31].

To investigate the role of the active compounds in SAL, we randomly divided the mice into 6 groups: control, ISO, ASA, Lut (luteolin), SYD (syringaldehyde), and COMB (luteolin and syringaldehyde). The basis of the drug dose settings was in line with previous studies and preliminary experiments [20, 32]. Mice in the treatment groups received Lut (25 mg/kg, CAS# 491-70-3, Shanghai Ye Yuan Biotechnology Co., Ltd, HPLC ≥ 98%) and SYD (50 mg/kg, CAS#134-96-3, Ye Yuan, HPLC ≥ 98%) and their mixture orally for 2 weeks-days. Finally, the mice were anaesthetized with sodium pentobarbital (60–70 mg/kg, i.p.), and cardiac function parameters were measured by three-lead electrocardiography (ECG). Serum was obtained by centrifuging blood samples, and heart tissues were collected for RT-qPCR and Western blot analysis. Additionally, freshly prepared hearts were fixed in 4% paraformaldehyde for 24 h and embedded in paraffin for histological analyses.

### Assay for markers of myocardial injury in the serum

Serum levels of cardiac troponin c(Tn-T) (Cat# XY9M0582), BNP (Cat# XY9M0877), LDH (Cat# XY9M437321) and ANP (Cat# XY9M0836) were determined using commercially available standard kits (Shanghai Xinyu Biotechnology Co., Ltd, China). The experiment was carried out on the basis of instructions provided by the manufacturers.

### Histology

The Trichrome Stain (Masson) Kit (Cat# G1340-7, Solaibao, China) was used to perform Masson's trichrome staining on 5 μm heart sections. Collagen fibers and nuclei appeared blue, while cytoplasm, muscle fibers, and red blood cells were red. Additionally, heart sections underwent examination with the HE staining kit (Cat#CD002A, Zhonghui Hecai Bio-pharmaceutical Technology Co., Ltd. and Cat# G1120, Solaibao) to assess heart tissue damage following the manufacturer's instructions. Subsequently, six random areas of each sample were observed under an Olympus BX51 fluorescence microscope. To measure cardiomyocyte cross-sectional area, FITC-conjugated wheat germ agglutinin (WGA) stained heart sections were employed. We first repaired antigens in paraffin sections by dewaxing and soaking them in citric acid antigen repair buffer (Cat# P0083, Beyotime, China). The sections were then incubated with WGA-FITC (5 μg/ml in PBS, GeneTex, USA) overnight in the dark at room temperature. Fluorescence quenching sealing tablets were used and images were acquired and analyzed with Image J software.

### Western blot

Western blot analyses were performed on cardiac tissue samples. Total proteins were obtained by RIPA Lysis Buffer (Cat.No. P0013B, Beyotime, China). Proteins were transferred to PVDF membranes using standard protocols after separation by SDS-PAGE. Subsequently, the membranes were incubated with rabbit monoclonal antibody overnight at 4 °C after blocking. Detection of the membranes was done using a chemiluminescence imaging system (ChemiDoc XRS + , BIO-RAD) following incubation with secondary antibodies (1:10000, ab6721, Abcam). The following antibodies used for the experiments are listed in Table S1, and quantitative analysis was performed using Image J.

### Quantitative Real-Time PCR (qRT-PCR)

Total RNA was extracted from the tissues with the Takara mini kit (No.9767, Takara), and the concentration and purity of the RNA were measured with the Ultra-micron Nucleic Acid protein analyzer (ThermoND2000). After reverse transcription using the PrimeScript RT Reagent Kit with gDNA Eraser (Cat.No. RR047A, Takara) according to the manufacturer's instructions, the gene expression was tested using a Green TM Premix Ex TaqTMII (CAT# RR820A, Takara) on the ABI StepOnePlus^™^ Real-Time PCR System with Tower. The results have been carried out with 3 replicates of each of the samples. The fold change in the expression of the genes was calculated by application of the 2^−ΔΔCt^ method. The values are expressed relative to *Gapdh* mRNA. Table 1 shows the PCR primers used for the analysis.

**Table 1:** Sequences of the primers for the genes used in the qRT-PCR assays

Gene	Spcies	Forward primer	Reverse primer
*Gapdh*	Mouse	AAGAAGGTGGTGAAGCAGGCATC	CGGCATCGAAGGTGGAAGAGTG
*Anp*	Mouse	TCGTCTTGGCCTTTTGGCT	TCCAGGTGGTCTAGCAGGTTCT
*Pi3kcg*	Mouse	AGTGTGGCTGCGGAGTTCTACC	TAACCAGACGGCGGCGAGTG
*Sod*	Mouse	AACCAGTTGTGTTGTCAGGAC	CCACCATGTTTCTTAGAGTGAGG
*Cat*	Mouse	AGCGACCAGATGAAGCAGTG	TCCGCTCTCTGTCAAAGTGTG

### Statistical analysis

The Statistical results are shown as mean ± standard deviation. To determine *P*-values, statistical analysis was carried out by means of the Student's t-test or two-way analysis of variance (ANOVA) by applying GraphPad Prism 8. Significance was defined as a *P* value of less than or equal to 0.05.

## Results

### SAL ameliorates cardiac damage in ISO-induced heart failure

To investigate whether SAL led to cardiac function recovery after ISO stimulation, we detected heart function indicators and heart histology. We found that cardiac hypertrophy in mice treated with SAL is apparent relative to control, as evidenced by marked elevations of heart-to-body weight and lung wet/dry weight ratio (*P* < 0.05) (Fig. 2B, D, E. Notably, the lung wet-to-dry weight ratio also serves as an indicator of cardiac failure [33]. ISO-induced changes were effectively inhibited by SAL treatment in mice (Fig. 2C). More importantly, following SAL treatment, the specific markers levels of cardiac injury including BNP, TNT, AST and ANP normalized in serum and tissue of mice (Fig. 2F, G). Overall, these findings indicate that SAL can attenuate heart damage by ISO administration.

### Potential components and targets of SAL

**Fig. 3:**
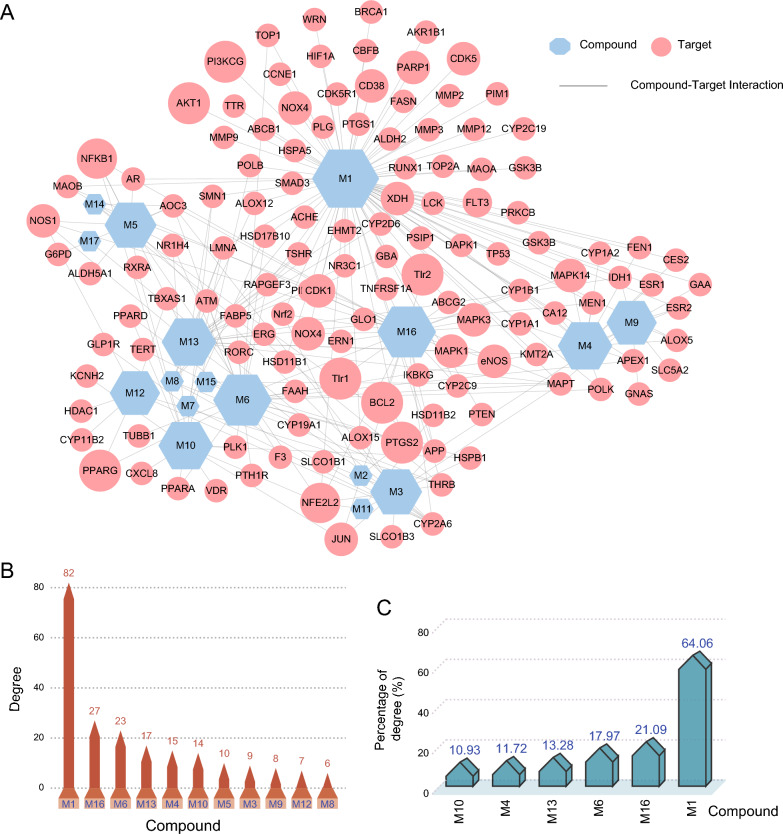
The Compound-target (C-T) network analysis. **A** The C-T network. **B** The degree of the active ingredients in SAL. **C** Percentage of active ingredient degree value. The red nodes correspond to targets, and the blue nodes to SAL’s active compounds. M1, luteolin; M16, syringaldehyde; M6, oleic acid

Favorable ADME properties are seen as a key driver for drug development. Utilizing three in silico pre-screening models, we identified 17 ingredients in SAL with desirable pharmacokinetic properties (DL ≥ 0.18, OB ≥ 30% or HL ≥ 4, methods), primarily consisting of volatile oils, flavonoid and other compounds (Table S2). Through a comprehensive approach, we gathered 128 potential targets for 17 candidate compounds, which were then used to construct a Compound-Target (C-T) network. The network, illustrated in Fig. 3A–C, comprised 145 nodes and 227 edges, revealing that compounds from SAL interact with multiple targets, and each target is influenced by various compounds. Notably, 7 compounds exhibited high degrees (degree ≥ 10), indicating the multi-target nature within SAL. Among them, there were 82 (64.06%) targets of luteolin (M1, Lut), 27 (21.09%) of syringaldehyde (M16, SYD) and 23 (17.97%) of oleic acid (M6), demonstrating that they are critical roles in the network (Table S2 and Fig. 3C). Simultaneously, many targets track multiple active ingredients, such as NF-κB and Nrf2. Indeed, SAL could activate the SKN-1/Nrf2 signaling pathway for neuroprotection and aging retardation [34]. The cardioprotective role of Lut is intricately linked to the activation of the PI3K/AKT/Nrf2 signaling cascade [35]. Additionally, emerging research corroborates that the PI3K/AKT and YAP signaling axes are pivotal in enhancing cardiomyocyte proliferation and augmenting cellular survival mechanisms [36]. And SYD supplementation might reduce oxidative stress and inflammation in heart attack patients [37].

### Therapeutic effect of active ingredients of SAL on HF

To further identify the most promising combinations of compounds for the treatment of HF in SAL, we employed the probabilistic ensemble approach (PEA) to investigate and analyze drug combinations (Table 2). The findings revealed that the most promising compound combinations were luteolin and syringaldehyde (Lut + SYD, 0.95), luteolin and isorhamnetin (Lut + ISOR, 0.92), and luteolin and isovitexin (Lut + ISOV, 0.87), respectively. Notably, Lut and SYD exhibited the highest degree values in the C-T network analysis, suggesting a potentially strong synergistic effect in the treatment of HF.

**Table 2:** Top3 drug combinations with best synergistic combination among SAL compounds

Compound 1	Compound 2	Synergy probility
Luteolin	Syringaldehyde	0.95
Luteolin	Isorhamnetin	0.92
Luteolin	Isovitexin	0.87

**Fig. 4:**
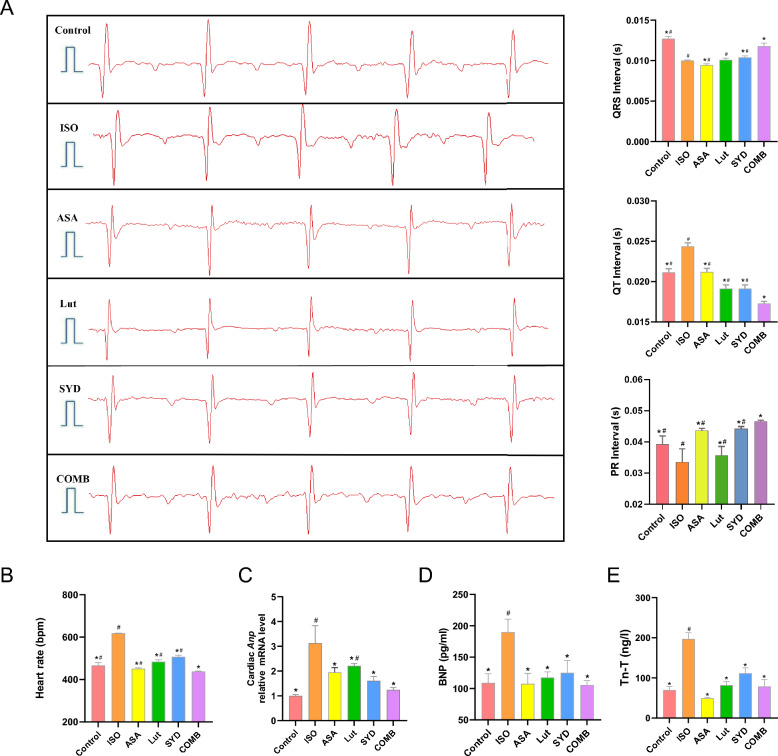
Therapeutic effect of active ingredients of SAL on the ISO-induced mouse model of HF. **A** ECG parameters assessment for various groups. **B** Heart rate, BPM: beats per minute. **C** Cardiac *Anp* relative mRNA level. **D** and **E** Serum levels of BNP and Tn-T measured in mice subjected to three different groups. *P < 0.05 compared to ISO group, #*P* < 0.05 compared to COMB group

To validate the effect of Lut and SYD of SAL in HF, an ISO-induced HF mouse model was utilized. Initially, ECG parameters were analyzed in the different groups to assess heart function. As illustrated in Fig. 4A, B, ECG parameters normalized after Lut, SYD and COMB treatment including the heart rate (HR) and PR interval in mice (*P* < 0.05). Interestingly, QT intervals, a hallmark of HF, were considerably prolonged in the ISO group but reversed in the drug-treated groups. In addition, the QRS interval was lower in the ISO group compared to the control group. After treatment with ingredients of SAL, the QRS interval was prolonged (*P* < 0.05), possibly due to alterations in resting membrane potential and the deceleration and amplitude of 0-phase and 1-phase conduction in cardiac myocytes during myocardial ischemia [38]. Importantly, the COMB group exhibited superior therapeutic effect compared to individual drug treatments (Fig. 4A). To further investigate how the active ingredients in SAL might potentially affect heart function, serum and heart tissue expression levels of BNP, c(TnT) and *Anp*, known heart dysfunction biomarkers, were measured (Fig. 4C–E). As anticipated, the TnT, BNP and *Anp* levels were elevated in the ISO group but suppressed by SYD and Lut treatment (*P* < 0.05).

**Fig. 5:**
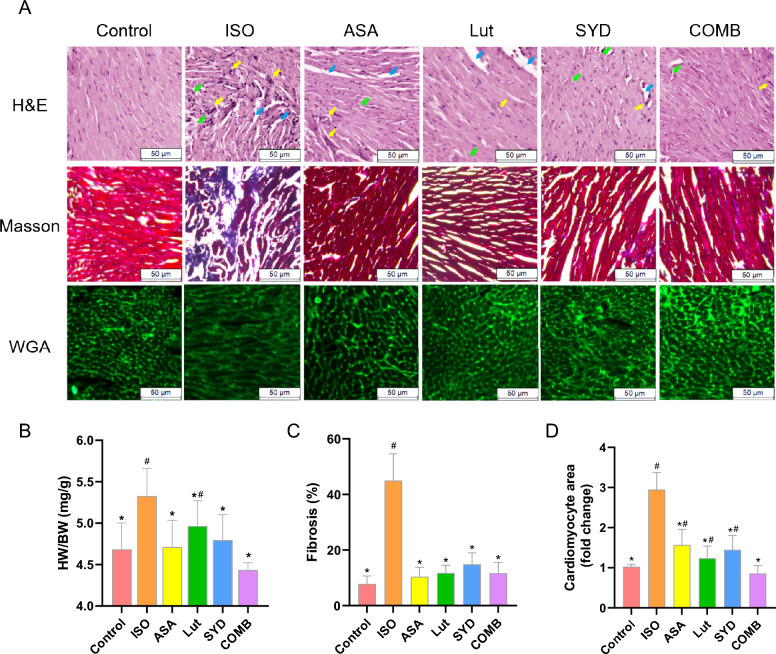
Representative histological results of the heart tissue staining. **A** H&E, Masson trichrome and wheat germ agglutinin (WGA) stains. Scale bar 50 μm. H&E staining, blue arrow: interstitial space of the myocardium. **B** HW/BW. **C** Quantification of the areas of cardiac fibrosis on sections stained with Masson's trichrome. **D** The cross-sectional area of cardiomyocytes (fold change) calculated from sections stained with WGA-FITC. #*P* < 0.05 in comparison with the COMB group, *P < 0.05 in comparison with the ISO group

HF is typically characterized by cardiac hypertrophy and fibrosis [39]. Then, HE, wheat germ agglutinin (WGA) and Masson stains were applied. WGA staining revealed that the increase in cardiomyocyte cross-sectional area induced by ISO was partially mitigated by Lut and SYD treatments (Fig. 5A, D). Similarly, the ratio of heart weight to body weight (HW/BW) was also markedly increased following the ISO intervention and showed a tendency towards normalization after Lut, SYD and COMB treatments (Fig. 5B). Meanwhile, we observed an increase in cardiac fibrosis area, infiltration of inflammatory cells and cardiomyocyte disorganization in the ISO group. Nonetheless, treatment with Lut and SYD reversed these effects (Fig. 5A, C). These findings suggest that the active ingredients of SAL could alleviate myocardial fibrosis and inflammation in the hearts of mice with HF.

### Biological mechanism analysis of SAL

**Fig. 6:**
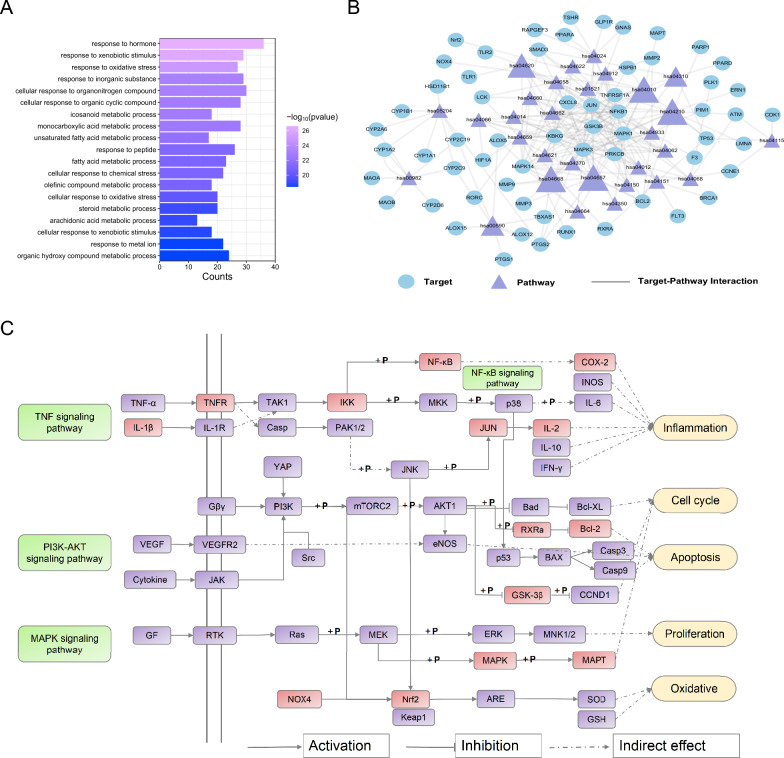
Biological mechanism analysis of SAL. **A** GOBP analysis. **B** the Target-Pathway (T-P) network. Potential targets and the KEGG pathways are represented by blue and purple nodes, respectively. And edges between purple and blue nodes suggest the destination on that pathway. **C** The representative HF pathway and therapeutic modules of SAL. Purple rectangles represent the targets of active ingredients in SAL, red rectangles represent the targets of HF pathway, respectively

To delve deeper into the biological mechanisms driving the therapeutic effects of SAL, the functional enrichment analysis was carried out. Several functional targets were significantly enriched, mainly in regulation of oxidative stress, apoptotic process and inflammatory-related response, including arachidonic acid metabolic process and interleukin-4 and interleukin-13 signaling, as shown in Fig. 6A and Table S3*.* Previous evidence indicated that impairment of oxidative stress, inflammatory response and apoptosis are closely associated with HF [40].

The KEGG enrichment analysis revealed 30 significantly enriched pathways (*P* < 0.01) that are likely key pathways crucial in HF (Table 3). To further analyze the underlying key mechanisms influenced by the active compounds of SAL, we constructed the target-pathway (T-P) network. The T-P network, depicted in Fig. 6B, consists of 90 nodes (60 targets and 30 pathways) and 223 edges. Notably, all 60 targets (60/60) are involved in multiple pathways, indicating that SAL interacts with various signaling pathways associated with heart disease. Simultaneously, 14 out of the 30 pathways are modulated by multiple targets (degree ≥ 8), potentially representing critical mechanism of SAL in treating HF. Furthermore, the MAPK signaling pathway (hsa04010, degree = 13), PI3K-AKT signaling pathway (hsa04151, degree = 8), TNF signaling pathway (hsa04668, degree = 10), and Apoptosis (hsa04210, degree = 12) are highlighted as major target protein-linked pathways in Table 3.

**Table 3:** The information of disease-related pathway of SAL

Term	Discription	Degree
hsa04010	MAPK signaling pathway	13
hsa04210	Apoptosis	12
hsa04659	Th17 cell differentiation	12
hsa04933	AGE-RAGE signaling pathway in diabetic complications	12
hsa04657	IL-17 signaling pathway	11
hsa04668	TNF signaling pathway	10
hsa04024	cAMP signaling pathway	9
hsa04620	Toll-like receptor signaling pathway	9
hsa04151	PI3K-Akt signaling pathway	8
hsa04621	NOD-like receptor signaling pathway	8
hsa00982	Drug metabolism—cytochrome P450	8
hsa05204	Chemical carcinogenesis	8
hsa04660	T cell receptor signaling pathway	8
hsa00590	Arachidonic acid metabolism	8

In consideration of complicated mechanisms of SAL for the therapy of HF, we integrated the KEGG and T-P network analysis to build a comprehensive HF pathway. In the HF-integrated pathway, the active compounds in SAL target approximately 74% of the proteins (40/54) (Fig. 6C). SAL impacts HF by acting on multiple pathways that primarily regulate biological processes such as inflammation, oxidative stress, and apoptosis (Fig. 6C).

### SAL attenuates ISO-induced oxidative stress, apoptosis and inflammation

**Fig. 7:**
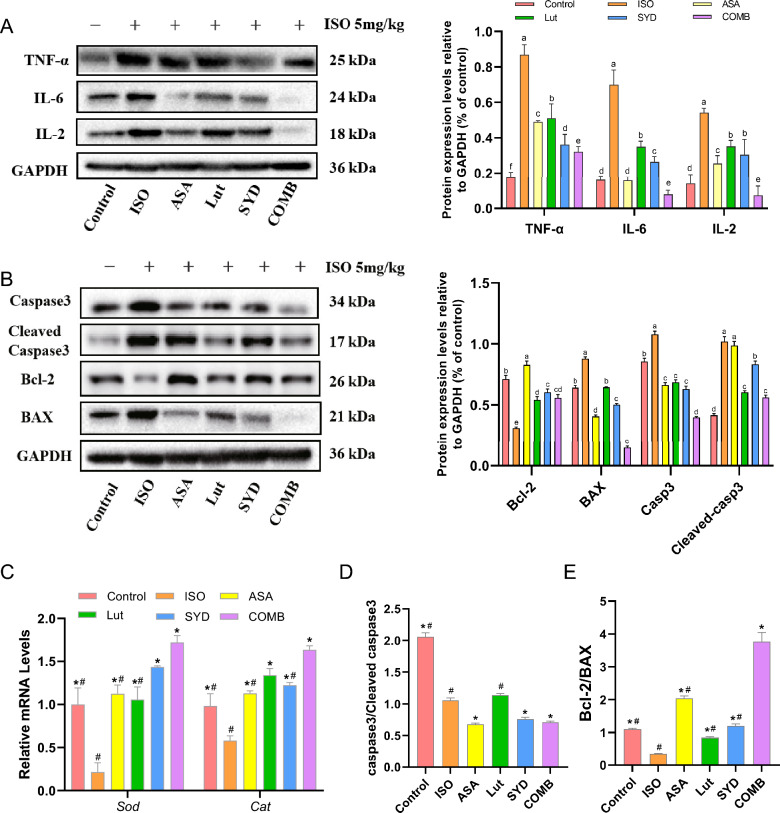
Functional enrichment experiment verification. **A** and **B** IL-2, IL-6, TNF-α, Cleaved-caspase3, caspase3, Bcl2 and BAX protein levels. **C** mRNA levels of *Sod* and *Cat* were measured in vivo. **D** The ratio of caspase3 to Cleaved caspase3. **E** Bcl-2/BAX ratio. Significant differences between groups marked with different letters. #*P* < 0.05 in comparison with the COMB group, *P < 0.05 in comparison with the ISO group

To determine whether the active compounds of SAL can inhibit inflammation and apoptosis and regulate ROS production, the expression of the relevant proteins or genes was determined by Western blot and RT-qPCR. Following drug treatment, a notable reduction in factors such as IL-6, TNF-α, and IL-2 was observed in heart tissue compared to the ISO group, suggesting a potential anti-inflammatory impact of the treatment (Fig. 7A) (*P* < 0.05). Moreover, the levels of the pro-apoptotic proteins cleaved caspase3 and Bax, as well as the anti-apoptotic protein Bcl-2 (B-cell lymphoma 2), were evaluated in cardiac tissue. As expected, SYD and Lut treatment relieved ISO-induced cardiomyocyte injury, evidenced by reduced caspase-3 and cleaved caspase-3 levels and an increased Bcl-2/BAX ratio (Fig. 7B, D, E). Similarly, SYD and Lut treatment prevented the decrease in *Sod* and *Cat* mRNA levels induced by ISO (Fig. 7C) (*P* < 0.05 or *P* < 0.01), highlighting that the retention of endogenous antioxidants is responsible for the cardioprotective effect of SAL. Notably, the above results are most pronounced in the COMB group. Collectively, these data demonstrate that SAL exhibits not only antioxidant and anti-inflammatory properties but also exerts a significant anti-apoptotic effect.

### Effect of SAL on YAP and PI3K/AKT signaling pathway

**Fig. 8:**
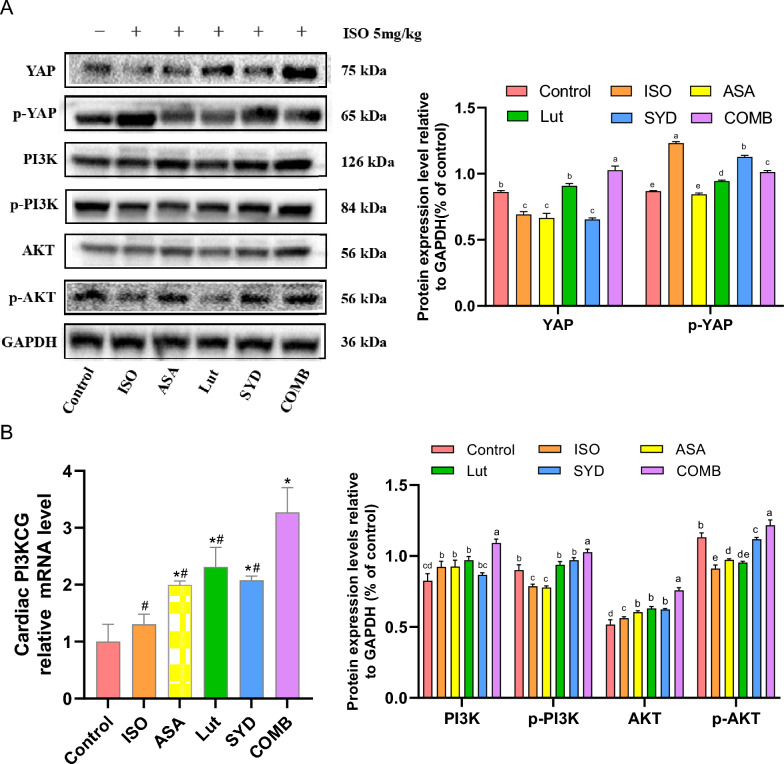
SAL’s effect on YAP and PI3K/AKT signaling pathway. **A** Detection and quantification of the expression of PI3K, p-PI3K, AKT, p-AKT, YAP and p-YAP proteins. YAP activation was detected by western blotting by observing the de-phosphorylation of YAP. **B** mRNA levels of PI3KCG in ventricular tissue of different groups. Groups marked with different letters were significantly different. The symbol # indicates a *P* value of less than 0.05 when compared with the COMB group, while the symbol * indicates a *P* value of less than 0.05 when compared with the ISO group

The implications of the systems pharmacology analysis were assessed by investigating the effects of the active compounds of SAL on key proteins in the integrated 'The HF pathway'. As shown in Fig. 8A, in the ISO group, YAP expression was significantly decreased in mouse heart tissue compared to control. The reduction was inhibited by treatment with SYD and Lut. Additionally, the levels of the p-PI3K and p-AKT proteins were downregulated in the ISO group but upregulated after drug treatment, indicating activation of the PI3K/AKT pathway (Fig. 8A, B). Together, these data imply that SAL might exert its cardiac therapy through YAP and PI3K-AKT pathway activation. YAP has been found to directly promote the coregulated transcription of Pik3cb, thereby leading to the activation of the PI3K/AKT pathway and the proliferation and survival of cardiomyocytes [36]. Overall, these results highlight potential therapeutic targets of SAL for HF.

## Discussion

HF is a severe condition with a high mortality rate characterized by an increased intrinsic heart rate [41]. Neurohormonal hyperactivation and extracellular matrix deposition are common features of this disease [42]. ISO induces cardiac remodeling, leading to pathological changes akin to those observed in human heart failure [43]. The mouse model created with ISO is a dependable tool for assessing the efficacy of drugs in treating hypertension and protecting the heart [44, 45]. The model is commonly employed to mimic HF in mice caused by stress-induced cardiomyopathy [46]. As expected, ISO-treated mice displayed typical HF pathology, including cardiac dysfunction, increased collagen deposition, and hypertrophic cardiomyocytes, consistent with previous research [47, 48]. These pathological abnormalities might be linked to inadequate oxygen supply and significantly elevated wall stress, suggesting a potential role of oxidative stress and inflammation in cardiac injury [49].

As a traditional Chinese medicine, SAL and its formula have been utilized in clinical settings for rheumatoid arthritis, angina and coronary artery disease [50]. The formulation 'Sanwei-Tanxiang powder' has an extensive clinical history to improve cardiac function and protect against heart damage caused by ISO [51]. Importantly, we have confirmed the cardioprotective properties of SAL in an ISO-induced HF model. Although SAL is commonly prescribed for cardiovascular conditions and demonstrates various pharmacological effects, further research is needed to explore the active ingredients and specific mechanisms of action responsible for its therapeutic effects.

Systems pharmacology, an emerging field, delves into the effects of drugs, ranging from the molecular scale to the whole organism. Our study employs a systems pharmacology approach to uncover the underlying mechanisms of SAL. We identified 17 candidates in SAL and their corresponding 128 potential cardiovascular disease targets through pharmacokinetic evaluation and target prediction. The average target count per compound was 7.53, demonstrating that SAL’s cardiovascular effects are multi-component and multi-targeted. Among them, Lut, SYD and isorhamnetin were the compounds with the highest degree values. Indeed, Lut was shown to ameliorate post-ischemic reperfusion injury by modulating the PI3K-AKT pathway and NF-κB pathways, thereby reducing the inflammatory response and mitigating oxidative stress [52]. Our pharmacological research also revealed that the active compounds of SAL significantly improved cardiac function parameters including HR, PR interval, QT interval and QRS interval in HF mice. While experimental constraints precluded the measurement of some cardiac function parameters by echocardiography, we endeavor to overcome these limitations in subsequent research endeavors. Treatment with SYD and Lut resulted in more organized cardiomyocytes with reduced collagen deposition and hypertrophy. These compounds also mitigated inflammatory cell infiltration and lowered ISO-induced serum c(TNT) and BNP levels. The combination of SYD and Lut exhibited a synergistic effect, enhancing ventricular function restoration and delaying pathological regression.

Network analysis identified SAL’s pivotal molecules, targets and pathways, revealing three main modules: oxidative stress, apoptosis and inflammation. SOD and CAT regulate oxidative stress as the first line of cellular defence [53]. ISO not only directly increases oxidative stress but also exacerbates cardiac hypoxia, leading to increased ROS production [54]. Antioxidant enzyme activity, such as SOD and CAT, decreased after ISO treatment in mice, while ASA, Lut, and SYD treatment increased their activity. In the COMB group, mice exhibited a marked increase in antioxidant enzyme activity, possibly due to COMB's role in protecting these enzymes from free radical damage.

Inflammation is strongly interfering with HF development [55]. It is primarily linked to cardiomyocyte hypertrophy, fibrosis, and ventricular remodeling, and even cardiomyocyte death [55]. Peripheral pro-inflammatory factors are notably elevated in cardiomyopathy patients and closely tied to the prognosis of the disease. Following treatment with ASA, Lut, SYD, and COMB, the expression of inflammatory factors decreased, thereby improving myocardial injury by suppressing inflammation. Decreased myocardial contractility and increased apoptotic cell count are common contributors to HF [56]. Apoptosis plays a crucial role in ventricular remodeling and HF progression. Lut, SYD, and COMB treatments increased Bcl-2 expression and decreased Cleaved-caspase 3 and BAX expression, indicating that the active components of SAL inhibit cardiomyocyte apoptosis.

H Chang et al. demonstrated that the PI3K/AKT pathway can inhibit myocardial apoptosis by phosphorylating AKT and enhancing the activity of Bcl-2, a molecule involved in the prevention of apoptosis [57]. YAP could protect heart muscle cells by diminishing the activity of apoptosis-related proteins including cleaved caspase 3 [58]. Furthermore, premature skin fibroblast senescence in diabetic mice was suppressed through activation of the PI3K/Akt/mTOR pathway and YAP nuclear translocation [59]. Enhanced expression of YAP, along with elevated levels of phosphorylated PI3K and AKT in the COMB group, suggested an activation of the PI3K-AKT signaling pathway.

In a nutshell, our study confirms that SAL improves HF by synergistically exerting anti-inflammatory, antioxidant, and anti-apoptotic effects. These results offer further valuable insights into the mechanisms of action of SAL in HF and suggest that SAL may hold promise as an herbal remedy for treating HF.

### Supplementary Information

Supplementary material 1.

## Data Availability

The data underlying this paper can be found in the paper as well as in its online supplementary material.

## Abbreviations

HF: Heart failure
SAL: *Santalum album* L.
Lut: Luteolin
SYD: Syringaldehyde
ISO: Isoproterenol
ASA: Aspirin
PEA: Probabilistic ensemble aggregation approach
C-T network: Compound-target network
T-P network: Target-pathway network
COMB: Combination
TCM: Traditional Chinese medicine
WES: Weighted ensemble similarity
SysDT: Systematic drug targeting tool
OB: Predict oral bioavailability
DL: Predict drug-likeness
HL: Predict half-life
HW/BW: Heart weight/body weight
