# Supplementary material for: Santalum album L. alleviates cardiac function injury in heart failure by synergistically inhibiting inflammation, oxidative stress and apoptosis through multiple components
Source: Chin Med. 2024 Jul 15;19:98. doi: 10.1186/s13020-024-00968-0 (PMC11251102; doi:10.1186/s13020-024-00968-0)
Supplement: Supplementary file 1 — Supplementary material 1. [file 13020_2024_968_MOESM1_ESM.docx]

Table S1 The primer antibodies information

| Antigen | Source | Dilution |
| --- | --- | --- |
| Anti-IL 6 | Abcam (ab229381) | 1:1000 |
| Anti-IL 2 | Abcam (ab92381) | 1:5000 |
| Anti- TNF-α | Abcam (ab183218) | 1:1000 |
| Anti-Bcl2 | Abcam (ab182858) | 1:2000 |
| anti-YAP | Abcam (ab205270) | 1:1000 |
| anti-phosphor-YAP | Abcam (ab76252) | 1:10000 |
| anti-AKT | Abcam (ab179463) | 1:10000 |
| anti-phosphor-AKT (T308) | Abcam (ab38449) | 1:500 |
| anti-PI3KCG | Abcam (ab32089) | 1:1000 |
| anti- phosphor-PI3KCG (Y607) | Abcam (ab182651) | 1:500 |
| anti-Bax | Abcam (ab32503) | 1:1000 |
| Anti-GAPDH | Abcam (ab181602) | 1:10000 |
| anti-Caspase-3 | Abcam (ab13847) | 1:500 |
| anti-Cleaved Caspase-3 | Abcam (ab32042) | 1:500 |

Table S2 The active compounds of SAL and their corresponding ADME parameters

| ID | MOL_ID | Compounds | Structure | OB | DL | HL | Degree |
| --- | --- | --- | --- | --- | --- | --- | --- |
| M1 | MOL000006 | luteolin | 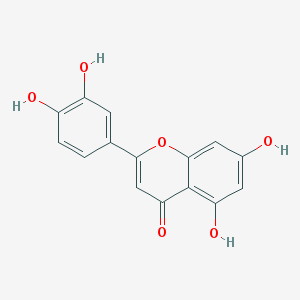 | 36.16 | 0.25 | 15.94 | 82 |
| M16 | MOL003177 | syringaldehyde | 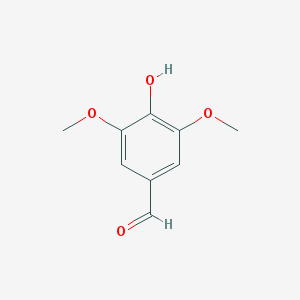 | 67.06 | 0.05 | 11.47 | 27 |
| M6 | MOL000675 | oleic acid | 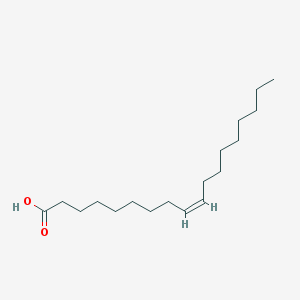 | 33.13 | 0.14 | 4.99 | 23 |
| M13 | MOL007581 | 4-aminopyridine | 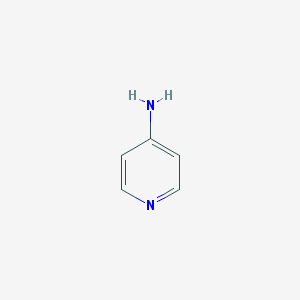 | 78.51 | 0.01 | 12.02 | 17 |
| M4 | MOL000354 | Isorhamnetin | 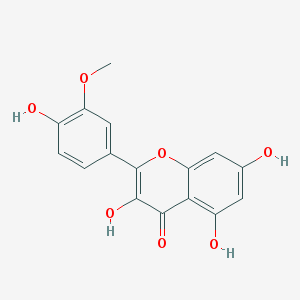 | 49.6 | 0.31 | 14.34 | 15 |
| M10 | MOL002850 | 2,6-Di-tert-butyl-4-methylphenol | 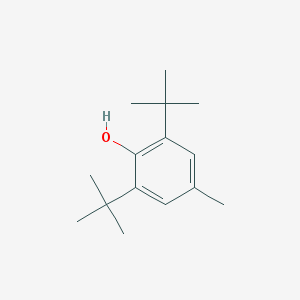 | 40.02 | 0.07 | 10.36 | 14 |
| M5 | MOL000635 | vanillin | 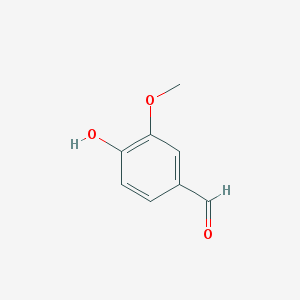 | 52 | 0.03 | 11.79 | 10 |
| M3 | MOL000119 | nerolidol | 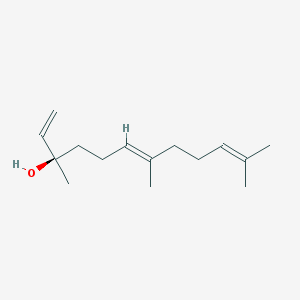 | 40.43 | 0.06 | 4.73 | 9 |
| M9 | MOL002322 | Isovitexin | 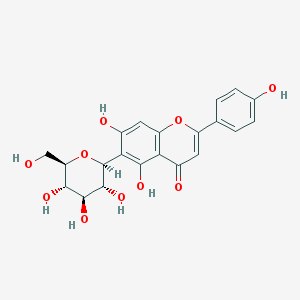 | 31.29 | 0.72 | 16.45 | 8 |
| M12 | MOL007580 | 3-methylpyridine | 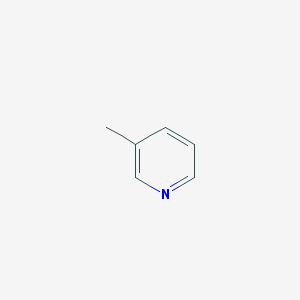 | 73.75 | 0.01 | 11.95 | 7 |
| M8 | MOL002031 | toluene | 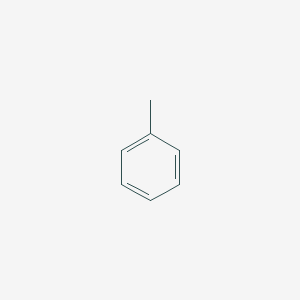 | 42.58 | 0.01 | 11.95 | 6 |
| M7 | MOL000775 | ethylacetate | 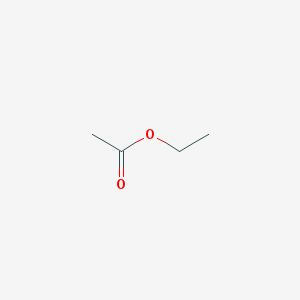 | 45.02 | 0 | 11.64 | 4 |
| M14 | MOL007545 | (z)-lanceol | 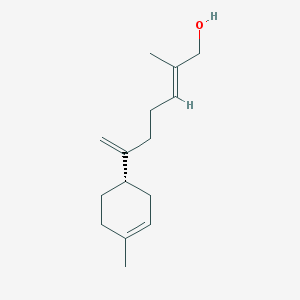 | 37.54 | 0.07 | 4.96 | 4 |
| M11 | MOL003557 | valencene | 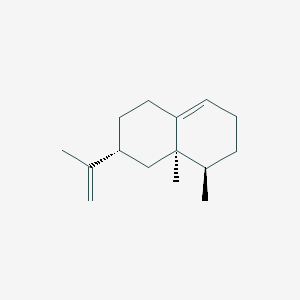 | 37.63 | 0.08 | 7.72 | 3 |
| M17 | MOL007595 | santenone | 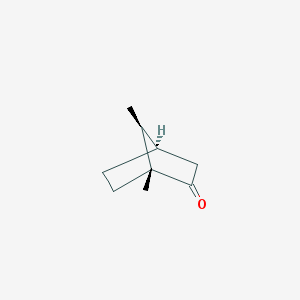 | 88.37 | 0.04 | 11.14 | 3 |
| M2 | MOL000023 | D-Limonene | 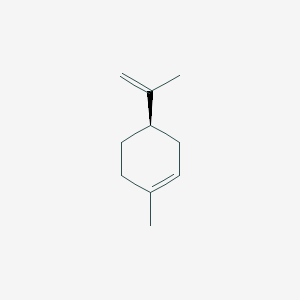 | 39.84 | 0.02 | 11.68 | 3 |
| M15 | MOL000302 | Undecanoic acid | 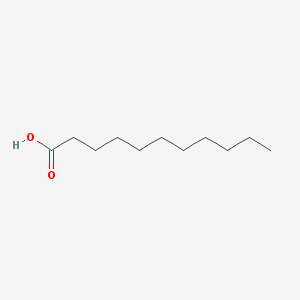 | 30.14 | 0.03 | 4.93 | 2 |

TableS3 The Gene Ontology (GO) biological process enrichment analysis on the potential targets

|  | Description | Count | Pvalue |
| --- | --- | --- | --- |
| 1 | response to hormone | 36 | 2.73057725635517E-27 |
| 2 | response to xenobiotic stimulus | 29 | 8.69817012220877E-27 |
| 3 | response to oxidative stress | 27 | 1.24953229494788E-25 |
| 4 | response to inorganic substance | 29 | 6.42314623359134E-24 |
| 5 | cellular response to organonitrogen compound | 30 | 6.65437774247401E-24 |
| 6 | cellular response to organic cyclic compound | 28 | 1.42940342435237E-23 |
| 7 | icosanoid metabolic process | 18 | 1.59387905997922E-23 |
| 8 | monocarboxylic acid metabolic process | 28 | 2.72146807049131E-23 |
| 9 | unsaturated fatty acid metabolic process | 17 | 9.20383428545424E-23 |
| 10 | response to peptide | 26 | 4.90887637380099E-22 |
| 11 | fatty acid metabolic process | 23 | 7.45503743710584E-22 |
| 12 | cellular response to chemical stress | 22 | 8.29659924203817E-22 |
| 13 | olefinic compound metabolic process | 18 | 1.11025323302882E-21 |
| 14 | cellular response to oxidative stress | 20 | 8.92294644946313E-21 |
| 15 | steroid metabolic process | 20 | 7.60206731032397E-20 |
| 16 | arachidonic acid metabolic process | 13 | 1.08952323901774E-19 |
| 17 | cellular response to xenobiotic stimulus | 18 | 1.83758570134642E-19 |
| 18 | response to metal ion | 22 | 3.28755895386902E-19 |
| 19 | organic hydroxy compound metabolic process | 24 | 3.29913559246919E-19 |
